# CSP and Epidemiology: a history of synergies and a future of challenges

**DOI:** 10.1590/0102-311XEN150924

**Published:** 2024-09-20

**Authors:** Guilherme Loureiro Werneck

**Affiliations:** 1 Instituto de Medicina Social, Universidade do Estado do Rio de Janeiro, Rio de Janeiro, Brasil.

*Cadernos de Saúde Pública* (CSP) completed 40 years of existence, which were marked by its commitment to quality, innovation, and plurality in scientific publication in Public Health. During its history, editorial policies have been improved to ensure gradual adherence to the best and most current principles of Open Science, as well as integrity and ethics in research and scientific publication. The articles published in CSP reveal the journal’s historical commitment to Public Health, including contributions from a variety of fields of knowledge and disciplines, which provided enormous services to the strengthening of research and scientific dissemination in this field.

Over these 40 years, many transformations have occurred in academic activities, of which the pages of CSP were part of the Brazilian Epidemiology history in this period. The journal gave visibility to the plurality of practices and approaches within Epidemiology, enabling the achievement of some of the objectives of this field, namely: to study the population distribution of health events and to clarify the determinants and limitations that lead to the heterogeneous distribution of such events in space and time ^1^. The dissemination of the results of these epidemiological studies is a cornerstone for reaching the Epidemiology goal: to subsidize public policies aimed at promoting health and improving the quality of life of populations, as well as reducing social inequities in health ^1^ ^,^ ^2^.

Over the years, CSP’s development has been followed by the expansion of Brazilian National Graduate Systems, with unquestionable effects on the training of professors and researchers and on the expansion and qualification of scientific production in many fields of knowledge ^3^. The expansion of academic graduate studies in Public Health from 1990 to 2022 is remarkable. In 1990, Public Health had only nine graduate programs, four of which were Master’s degrees and five combined Master’s and PhD degrees ^3^. In 2022, there were almost 100 graduate programs in 22 states, nearly all with areas of concentration and/or lines of research in Epidemiology. At this time, the graduate programs had almost 9,000 students enrolled or graduated, in addition to more than 2,000 active PhDs professors. Overall, such process of expansion of graduate programs Public Health certainly reflected in the expansion of the base of researchers and professors in Epidemiology, contributing to the growth and improvement of scientific production. Thus, it is reasonable to assume that the paths traced over the years by CSP and by the graduate studies in Epidemiology have led to mutual benefits, with the journal providing the qualified space for the dissemination of research in Epidemiology for the graduate programs, all together with the programs providing CSP with greater figures of qualified scientific production.

From 2001 to 2024, about 2,900 articles were evaluated and approved by Associate Editors of Epidemiology backgrounds at CSP. The aforementioned figure represents approximately 1/4 of all articles published in the journal in these years. Notably, these numbers represent only a relatively simplified estimate of the total number of articles in Epidemiology, since articles of an epidemiological nature may have been approved by editors not classified as epidemiologits and vice versa. In any case, this substantial number of articles reflects the strength of Epidemiology in the scientific production within CSP’s Public Health. It is not surprising that Epidemiology contributes with such volume of papers, given the very characteristics of its scientific production process, which finds in scientific articles its most typical vehicle for research outcomes dissemination. It is also noteworthy the greater institutionalization of epidemiological practices in health services, especially epidemiological surveillance, an important source of data and information on health.

To appreciate the contribution of Epidemiology published in CSP from 2001 to 2024, we analyzed keywords from 2,599 of these articles (89%) that presented abstracts and could be located by searching on the SciELO website (https://www.scielo.br/). Initially, we selected 4,858 of the first three keywords from the articles, excluding those that were repeated or that represented similar themes or research topics within a given article. After a process of harmonization of the terms, 4,495 keywords that had been used in at least two articles were classified into 41 themes. Note that, this classification was made freely by the author, according to his experience and worldview, without an explicit theoretical reference. Furthermore, one to three terms per article were analyzed, so that this approach provides a view of the selected keywords set as the unit of analysis, not the whole article. Thus, interpretations of these results should be cautiously made.

According to the classification used, the themes represented in more than 2% of the analyzed keywords were: “food and nutrition” (10.1%); “infectious diseases” (9.7%); “health status indicators and measures of disease occurrence” (7.4%); “epidemiology in health services” (6.4%); “child health” (4.4%); “noncommunicable diseases” (4.3%); “environmental and occupational health” (3.9%); “social determinants and inequalities in health” (3.1%); “adolescence and health” (3.1%); “aging” (3%); “care during pregnancy, childbirth, and puerperium” (3%); “sexual and reproductive health” (2.8%); “violence and accidents” (2.5%); “health surveillance and information systems” (2.3%); and “mental health” (2.3%). Regarding the study designs, surveys and cross-sectional studies were mentioned in 2.6% and cohort and case-control studies in 1.2% of the keywords analyzed. Statistical methods of data analysis were specified in 1.9%, while studies of risk factors comprised 2.5% of the total keywords. Among infectious diseases, HIV/AIDS, dengue, tuberculosis, Chagas disease, and leishmaniasis were the most cited. Other specific research topics deserve to be noted, namely: “smoking, alcohol, and drug consumption” (83 mentions); “physical activity” (69); “women’s health” (68); “oral health” (67); “quality of life” (55); and “immunization” (42). Studies on data collection instruments stand out, with 57 mentions, including an important range of studies evaluating validity and reliability.

If the study of so many themes offers some comfort that Epidemiology forms a thriving and diverse community, the absences are also glaring. Epidemiological studies that address pressing current issues should be further stimulated, namely: gender issues; racism; vulnerable populations such as Quilombolas, Indigenous people, ribeirinhos communities, and homeless people; human rights; people deprived of liberty; people with disabilities; preparedness and response to public health emergencies; climate crisis; misinformation; intersectionality; among many others. Thus, CSP should develop inclusive strategies to expand the share of studies on those issues in the journal. Such initiatives, especially if followed by effective actions by the graduation programs in Public Health, can contribute to mitigate the problem. However, since these are structural issues, which express the impacts of an inhumane and predatory economic model, it is necessary more than an Epidemiology-science, but an Epidemiology engaged in historical struggles to Public Health ^4^.

This profile allows us to visualize the scope of epidemiological investigations, captured here in the pages of CSP in over 20 years. Even considering the limitations of the employed approach, it is clear that the journal offers space for diversity in epidemiological investigations, from theoretical-methodological to thematic and operational studies. A formal bibliometric study, which uses quantitative and qualitative methods, accessing not only keywords, but also full abstracts and, eventually, interviews with authors and editors, may better reflect the role that CSP has played in the qualification of epidemiological research, while at the same time it may observe the possible benefits brought by Epidemiology to the CSP development. Throughout the journal existence, CSP has contributed to the dissemination of the Epidemiology research potential. However, for it to be carried out, we must leave our comfort zone and create new approaches to the investigation of emerging contemporary themes. In this challenge, Epidemiology continues to count on the support of CSP.

Long live CSP!
