# Supplementary material for: CSP and Epidemiology: a history of synergies and a future of challenges
Source: Cad Saude Publica. 2024 Sep 20;40(9):e00150924. doi: 10.1590/0102-311XEN150924 (PMC11415043; doi:10.1590/0102-311XEN150924)
Supplement: Translation

# Translation: CSP e a Epidemiologia: uma história de sinergias e um futuro de desafios

**DOI:** 10.1590/0102-311XPT150924

**Authors:** Guilherme Loureiro Werneck

**Affiliations:** 1 Instituto de Medicina Social, Universidade do Estado do Rio de Janeiro, Rio de Janeiro, Brasil.

*Cadernos de Saúde Pública* (CSP) completou 40 anos de existência, marcados pelo compromisso com a qualidade, inovação e pluralidade, na publicação científica em Saúde Coletiva. Nesse processo, políticas editoriais foram aprimoradas para garantir a adesão paulatina aos melhores e mais atuais princípios da Ciência Aberta e da integridade e ética na pesquisa e na publicação científica. Os artigos publicados em CSP revelam seu comprometimento histórico com a Saúde Coletiva, incluindo contribuições de uma variedade de áreas de conhecimento e disciplinas, prestando enormes serviços à consolidação da pesquisa e divulgação científica nesse campo.

Ao longo desses 40 anos, muitas transformações ocorreram nas atividades acadêmicas, e as páginas de CSP escrevem parte da história da Epidemiologia brasileira nesse período. CSP deu visibilidade à pluralidade de práticas e enfoques da Epidemiologia, permitindo a realização de uma parte de seus objetivos, quais sejam, estudar a distribuição populacional de eventos de saúde e esclarecer os determinantes e limitantes que levam à distribuição heterogênea desses eventos no espaço e tempo ^1^. A divulgação dos resultados dessas pesquisas epidemiológicas é parte fundamental para que a missão da Epidemiologia seja concluída, isto é, subsidiar políticas públicas que visem à promoção da saúde e a melhoria da qualidade de vida das populações, bem como a redução das iniquidades sociais na esfera da saúde ^1^ ^,^ ^2^.

O desenvolvimento de CSP, nesse período, foi acompanhado pela expansão do Sistema Nacional de Pós-Graduação, com reflexos decisivos na formação de docentes e pesquisadores e na ampliação e qualificação da produção científica em diferentes áreas do conhecimento ^3^. O crescimento da pós-graduação *stricto sensu* em Saúde Coletiva de 1990 a 2022 é notável. Em 1990, a área de Saúde Coletiva contava com apenas nove programas de pós-graduação, sendo quatro de mestrado e cinco combinando cursos de mestrado e doutorado ^3^. Neste ano, existiam quase 100 programas de pós-graduação em 22 estados, quase todos com áreas de concentração e/ou linhas de pesquisa em Epidemiologia. Nesse percurso de tempo, os programas de pós-graduação contavam com quase 9 mil alunos matriculados ou titulados e mais de 2 mil docentes doutores em atividade. Tal processo de expansão da pós-graduação em Saúde Coletiva certamente refletiu na ampliação da base de pesquisadores e docentes em Epidemiologia, contribuindo para o crescimento e qualificação da produção científica. Assim, é razoável supor que os caminhos traçados ao longo desses anos, por CSP e pela pós-graduação *stricto sensu* em Epidemiologia, levaram a benefícios mútuos, com CSP provendo o espaço qualificado para a veiculação de pesquisas em Epidemiologia para os programas de pós-graduação e, os programas fornecendo para CSP uma ampliação da produção científica qualificada.

Entre 2001 e 2024, cerca de 2.900 artigos foram avaliados e aprovados por Editores Associados de Epidemiologia de CSP. Isso representa, aproximadamente, 1/4 dos artigos publicados em CSP nesses anos, ressalvando-se que esses números representam apenas uma estimativa, relativamente simplificada, do total de artigos de Epidemiologia, já que artigos de cunho epidemiológico podem ter sido aprovados por editores não classificados como de Epidemiologia e vice-versa. De qualquer forma, tal número substancial de artigos reflete a força da Epidemiologia na configuração da produção científica em Saúde Coletiva em CSP. Não surpreende que a Epidemiologia apresente um volume expressivo de contribuições, dado às próprias características do seu processo de produção científica, que encontra em artigos científicos seu mais típico veículo de divulgação de resultados de pesquisa. Há de se salientar, também, a maior institucionalização das práticas epidemiológicas em serviços de saúde, em especial a vigilância epidemiológica, fonte importante de dados e informações em saúde.

Para apreciar algo da contribuição da Epidemiologia veiculada em CSP ao longo de 2001 a 2024, analisamos palavras-chave de 2.599 desses artigos (89%) que apresentavam resumo e puderam ser localizados em busca no *site* da base SciELO (https://www.scielo.br/). Inicialmente, selecionamos 4.858 entre as três primeiras palavras-chave apresentadas pelos artigos, excluindo-se aquelas repetidas ou que representassem temas ou tópicos de investigação similares dentro de um determinado artigo. Após um processo de harmonização dos termos utilizados, procedeu-se à classificação em 41 temas de 4.495 palavras-chave que haviam sido utilizadas em pelo menos dois artigos. Vale salientar que tal classificação foi realizada de forma livre pelo autor, de acordo com sua experiência e visão de mundo, sem uma referência teórica explícita. Ainda, foram analisados de um a três termos por artigo, de forma que a visão propiciada por essa abordagem tem como unidade de análise não o artigo, mas o conjunto de palavras-chave selecionadas. Assim, interpretações desses resultados devem ser feitas com cautela.

De acordo com a classificação utilizada, os temas representados em mais de 2% das palavras-chave analisadas foram “alimentação e nutrição” (10,1%), “doenças infecciosas” (9,7%), “indicadores de saúde e medidas de ocorrência de doenças” (7,4%), “epidemiologia em serviços de saúde” (6,4%), “saúde da criança” (4,4%), “doenças crônicas não-transmissíveis” (4,3%), “saúde ambiental e do(a) trabalhador(a)” (3,9%), “determinantes sociais e desigualdades em saúde” (3,1%), “adolescência e saúde” (3,1%), “envelhecimento” (3%), “cuidados na gestação, parto e puerpério” (3%), “saúde sexual e reprodutiva” (2,8%), “violência e acidentes” (2,5%), “vigilância em saúde e sistemas de informação” (2,3%) e “saúde mental” (2,3%). Em relação aos desenhos de estudo abordados, inquéritos e estudos seccionais foram citados em 2,6% e estudos de coorte e caso-controle em 1,2% das palavras-chave analisadas. Métodos estatísticos de análise de dados foram especificados em 1,9% e estudos de fatores de risco conformaram 2,5%. Entre as doenças infecciosas, HIV/aids, dengue, tuberculose, doença de Chagas e leishmanioses foram as mais citadas. Outros temas específicos de investigação merecem nota, como “tabagismo e consumo de álcool e drogas” (83 menções), “atividade física” (69 menções), “saúde da mulher” (68 menções), “saúde bucal” (67 menções), “qualidade de vida” (55 menções) e “imunização” (42 menções). Destacam-se os estudos sobre instrumentos de coleta de dados, com 57 menções; entre eles, uma gama importante de estudos de avaliação de validade e confiabilidade.

Se a presença de tantos temas oferece algum conforto de que a Epidemiologia conforma uma comunidade pujante e diversa, as ausências são também gritantes. Estudos epidemiológicos que abordam assuntos atuais prementes, como questões de gênero; racismo; populações vulnerabilizadas, por exemplo quilombolas, indígenas, ribeirinhos e moradores de rua; direitos humanos; população privada de liberdade; pessoas com deficiência; preparação e resposta a emergências em saúde pública; crise climática; desinformação; interseccionalidade; entre tantas outras, precisam ser estimulados. Para CSP fica a sugestão de desenvolver estratégias inclusivas para ampliar a participação desses estudos na revista. Tais iniciativas, principalmente se acompanhadas por ações efetivas dos programas de pós-graduação em Saúde Coletiva, podem contribuir para mitigar o problema. Entretanto, por se tratar de questões estruturais que expressam os impactos de um modelo econômico desumano e predatório, é necessário mais do que uma Epidemiologia-ciência, mas uma Epidemiologia engajada em lutas que são caras e históricas à Saúde Coletiva ^4^.

O perfil apresentado permite visualizar a abrangência das investigações epidemiológicas, aqui captadas nas páginas de CSP em pouco mais de 20 anos. Mesmo considerando as limitações da abordagem empregada, percebe-se que CSP oferece espaço para uma diversidade de investigações epidemiológicas, desde estudos teórico-metodológicos até estudos temáticos e operacionais. Um estudo bibliométrico formal, que utilize métodos quantitativos e qualitativos, acessando não somente palavras-chave, mas os textos dos resumos e, eventualmente, entrevistas com autores e editores poderá refletir melhor o papel que CSP tem desempenhado para a qualificação da pesquisa epidemiológica, ao mesmo tempo que possibilitará observar os eventuais benefícios gerados pela epidemiologia para o desenvolvimento de CSP. Ao longo de sua existência, CSP tem contribuído para que a pesquisa em Epidemiologia expresse sua potência, mas para que isso se realize é necessário que saiamos de nosso espaço de conforto e criemos abordagens para a investigação de temas contemporâneos emergentes. Nesse desafio, a Epidemiologia continua contando com o apoio de CSP.

Vida longa a CSP!

# Translation: CSP y la Epidemiología: una historia de sinergias y un futuro de desafíos

**DOI:** 10.1590/0102-311XES150924

**Authors:** Guilherme Loureiro Werneck

**Affiliations:** 1 Instituto de Medicina Social, Universidade do Estado do Rio de Janeiro, Rio de Janeiro, Brasil.

*Cadernos de Saúde Pública* (CSP) ha cumplido 40 años de existencia marcados por su compromiso con la calidad, la innovación y la pluralidad en la publicación científica en Salud Colectiva. En este proceso, se mejoraron las políticas editoriales para asegurar la adhesión gradual a los mejores y más actuales principios de la Ciencia Abierta y de la integridad y ética en la investigación y en la publicación científica. Los artículos publicados en CSP revelan su compromiso histórico con la Salud Colectiva, incluyendo aportes de una variedad de áreas del conocimiento y disciplinas, brindando enormes servicios a la consolidación de la investigación y a la divulgación científica en este campo.

A lo largo de estos 40 años, ocurrieron muchas transformaciones en las actividades académicas, y las páginas del CSP escriben parte de la historia de la Epidemiología brasileña en ese período. CSP ha dado visibilidad a la pluralidad de prácticas y enfoques de la Epidemiología, permitiendo la consecución de una parte de sus objetivos, a saber, estudiar la distribución poblacional de los eventos de salud y aclarar los factores determinantes y limitantes que conducen a la distribución heterogénea de estos eventos en el espacio y tiempo ^1^. La divulgación de los resultados de estas investigaciones epidemiológicas es parte fundamental para que se cumpla la misión de la Epidemiología: apoyar políticas públicas encaminadas a promover la salud y mejorar la calidad de vida de las poblaciones y reducir las inequidades sociales en salud ^1^ ^,^ ^2^.

El desarrollo de CSP a lo largo de estos años estuvo acompañado de la expansión del Sistema Nacional de Posgrado brasileño, con impactos decisivos en la formación de docentes e investigadores y en la ampliación y cualificación de la producción científica en diferentes áreas del conocimiento ^3^. El crecimiento de los estudios de posgrado estricto sensu en Salud Colectiva de 1990 al 2022 es notable. En 1990, el área de la Salud Colectiva contaba solo con nueve programas de posgrado, cuatro de los cuales de Maestría y cinco combinando cursos de Maestría y Doctorado ^3^. En el 2022, fueron casi 100 programas de posgrado en 22 estados, casi todos con áreas de concentración o líneas de investigación en Epidemiología. En ese momento, los programas de posgrado contaban con casi 9.000 estudiantes matriculados o titulados y más de 2.000 docentes con doctorado en actividad. Este proceso de expansión del programas de posgrado en Salud Colectiva en general, sin duda, se reflejó en la ampliación de la base de investigadores y docentes en Epidemiología, contribuyendo al crecimiento y a la cualificación de la producción científica. Así, es razonable suponer que los caminos trazados a lo largo de estos años por CSP y por el posgrado estricto sensu en Epidemiología, condujeron a beneficios mutuos, con CSP proporcionando el espacio cualificado para la circulación de investigaciones en Epidemiología para los programas de posgrado y los programas de posgrado brindando a CSP una ampliación de la producción científica cualificada.

Entre el 2001 y el 2024, los Editores Asociados de Epidemiología de CSP evaluaron y aprobaron alrededor de 2.900 artículos. Esto representa aproximadamente 1/4 de los artículos publicados en CSP en estos años, resaltando que estos números solo representan una estimación, relativamente simplificada, del número total de artículos de Epidemiología, ya que los artículos de naturaleza epidemiológica pueden haber sido aprobados por editores no clasificados como de Epidemiología y viceversa. De todos modos, este importante número de artículos refleja la fuerza de la Epidemiología en la configuración de la producción científica en Salud Colectiva en CSP. No es de extrañar que la Epidemiología presente un volumen importante de contribuciones, dadas las propias características de su proceso de producción científica, que encuentra en los artículos científicos su vehículo más típico de difusión de los resultados de la investigación. Cabe resaltar, aun, la mayor institucionalización de las prácticas epidemiológicas en servicios de salud, en especial la vigilancia epidemiológica, una fuente importante de datos e información en salud.

Para apreciar algo de la contribución de la Epidemiología difundida en CSP a lo largo del 2001 al 2024, analizamos palabras claves de 2.599 de estos artículos (89%) que presentaban un resumen y podían localizarse mediante una búsqueda en el sitio *web* de SciELO (https://www.scielo.br/). Inicialmente, seleccionamos 4.858 entre las tres primeras palabras claves presentadas por los artículos, excluyendo aquellas que se repetían o que representaban temas o tópicos de investigación similares dentro de un artículo determinado. Tras un proceso de armonización de los términos utilizados, se procedió a la clasificación en 41 temas de 4.495 palabras claves que habían sido utilizadas en al menos 2 artículos. Cabe señalar que esta clasificación fue realizada de forma libre por el autor, según su experiencia y cosmovisión, sin un referente teórico explícito. Además, se analizaron de 1 a 3 términos por artículo, de modo que la visión que proporciona este enfoque tiene como unidad de análisis no el artículo, sino el conjunto de palabras claves seleccionadas. Por lo tanto, las interpretaciones de estos resultados deben hacerse con cautela.

Según la clasificación utilizada, los temas representados en más del 2% de las palabras claves analizadas fueron “alimentación y nutrición” (10,1%), “enfermedades infecciosas” (9,7%), “indicadores de salud y medidas de incidencia de enfermedades” (7,4%), “epidemiología en servicios de salud” (6,4%), “salud del niño” (4,4%), “enfermedades crónicas no transmisibles” (4,3%), “salud ambiental y del(de la) trabajador(a)” (3,9%), “determinantes sociales y desigualdades en salud” (3,1%), “adolescencia y salud” (3,1%), “envejecimiento” (3%), “cuidados durante el embarazo, parto y puerperio” (3%), “salud sexual y reproductiva” (2,8%), “violencia y accidentes” (2,5%), “vigilancia en salud y sistemas de información” (2,3%) y “salud mental” (2,3%). En cuanto a los diseños de estudio cubiertos, las encuestas y estudios seccionales fueron citados en un 2,6% y los estudios de cohorte y de casos-control en un 1,2% de las palabras claves analizadas. Los métodos estadísticos de análisis de datos se especificaron en el 1,9% y los estudios de factores de riesgo representaron el 2,5% del total de palabras claves analizadas. Entre las enfermedades infecciosas, las más citadas fueron el VIH/sida, el dengue, la tuberculosis, la enfermedad de Chagas y la leishmaniasis. Otros temas específicos de investigación merecen mención, como “tabaquismo, consumo de alcohol y drogas” (83 menciones), “actividad física” (69), “salud de la mujer” (68), “salud bucal” (67), “calidad de vida” (55) e “inmunización” (42). Se destacan los estudios sobre instrumentos de recopilación de datos, con 57 menciones, incluyendo una importante gama de estudios de evaluación de validez y confiabilidad.

Si la presencia de tantos temas ofrece cierto consuelo de que la Epidemiología conforma una comunidad vibrante y diversa, las ausencias también son evidentes. Se deben fomentar estudios epidemiológicos que aborden temas apremiantes de la actualidad como cuestiones de género, racismo, poblaciones vulnerables, como quilombolas, indígenas, ribereños y personas en situación de calle, derechos humanos, población privada de libertad, personas con discapacidad, preparación y respuesta a emergencias en salud pública, crisis climática, desinformación, interseccionalidad, entre muchas otros. Para CSP queda la sugerencia de desarrollar estrategias inclusivas para aumentar la participación de estos estudios en la revista. Este tipo de iniciativas, especialmente si van acompañadas de acciones efectivas de los programas de posgrado en Salud Colectiva, puede contribuir a mitigar el problema. Sin embargo, dado que se trata de cuestiones estructurales, que expresan los impactos de un modelo económico inhumano y depredador, se necesita más que una Epidemiología-ciencia, sino una Epidemiología comprometida con las luchas que son valiosas e históricas para la Salud Colectiva ^4^.

El perfil presentado permite visualizar el alcance de las investigaciones epidemiológicas, plasmadas aquí en las páginas de CSP en poco más de 20 años. Incluso teniendo en cuenta las limitaciones del enfoque empleado, está claro que CSP ofrece espacio para una diversidad de investigaciones epidemiológicas que incluyen desde estudios teórico-metodológicos hasta temáticos y operativos. Un estudio bibliométrico formal, que utilice métodos cuantitativos y cualitativos, accediendo no solo a palabras claves, sino también a los textos de los resúmenes y, eventualmente, a entrevistas con autores y editores, podrá reflejar mejor el papel que ha desempeñado la CSP en la cualificación de la investigación epidemiológica, a la vez que podrá observar los posibles beneficios aportados por la Epidemiología al desarrollo de CSP. A lo largo de su existencia, CSP ha contribuido a que la investigación en Epidemiología exprese su poder, pero para que esto se realice es necesario que salgamos de nuestra zona de confort y creemos nuevos enfoques para la investigación de temas contemporáneos emergentes. En este desafío, la Epidemiología sigue contando con el apoyo de CSP.

¡Larga vida a CSP!
